# Role of deformable cancer cells on wall shear stress-associated-VEGF secretion by endothelium in microvasculature

**DOI:** 10.1371/journal.pone.0211418

**Published:** 2019-02-22

**Authors:** Mahsa Dabagh, Amanda Randles

**Affiliations:** Department of Biomedical Engineering, Duke University, Durham, North Carolina, United States of America; University of California San Diego, UNITED STATES

## Abstract

Endothelial surface layer (glycocalyx) is the major physiological regulator of tumor cell adhesion to endothelium. Cancer cells express vascular endothelial growth factor (VEGF) which increases the permeability of a microvessel wall by degrading glycocalyx. Endothelial cells lining large arteries have also been reported, *in vitro and in vivo*, to mediate VEGF expression significantly under exposure to high wall shear stress (WSS) > 0.6 Pa. The objective of the present study is to explore whether local hemodynamic conditions in the vicinity of a migrating deformable cancer cell can influence the function of endothelial cells to express VEGF within the microvasculature. A three-dimensional model of deformable cancer cells (DCCs) migrating within a capillary is developed by applying a massively parallel hemodynamics application to simulate the fluid-structure interaction between the DCC and fluid surrounding the DCC. We study how dynamic interactions between the DCC and its local microenvironment affect WSS exposed on endothelium, under physiological conditions of capillaries with different diameters and flow conditions. Moreover, we quantify the area of endothelium affected by the DCC. Our results show that the DCC alters local hemodynamics in its vicinity up to an area as large as 40 times the cancer cell lateral surface. In this area, endothelium experiences high WSS values in the range of 0.6–12 Pa. Endothelial cells exposed to this range of WSS have been reported to express VEGF. Furthermore, we demonstrate that stiffer cancer cells expose higher WSS on the endothelium. A strong impact of cell stiffness on its local microenvironment is observed in capillaries with diameters <16 μm. WSS-induced-VEGF by endothelium represents an important potential mechanism for cancer cell adhesion and metastasis in the microvasculature. This work serves as an important first step in understanding the mechanisms driving VEGF-expression by endothelium and identifying the underlying mechanisms of glycocalyx degradation by endothelium in microvasculature. The identification of angiogenesis factors involved in early stages of cancer cell-endothelium interactions and understanding their regulation will help, first to develop anti-angiogenic strategies applied to diagnostic studies and therapeutic interventions, second to predict accurately where different cancer cell types most likely adhere in microvasculature, and third to establish accurate criteria predisposing the cancer metastasis.

## 1. Introduction

Adhesion of deformable cancer cells (DCCs) to vascular endothelium is a critical stage of metastasis that precedes the invasion and extravasation of DCC [1–3]. Adhesive interactions between the DCC and endothelium, which serves as far more than a simple physical barrier between blood and interstitial fluid, are initiated by the loss of endothelial surface glycocalyx layer [3–6]. It has previously shown that DCC facilitates its own adhesion to the endothelium by secreting vascular endothelial growth factor (VEGF) that locally degrades the glycocalyx layer, resulting in the exposure of endothelial adhesion molecules [1–5]. It has also been reported that the endothelium acts as a promoter for adhesion of DCCs, but whether the endothelium contributes to the degradation of glycocalyx still remains elusive. Wall shear stress (WSS), the hemodynamic traction force, has been shown, *in vivo*, to contribute in VEGF expression by endothelial cells in the range of physiological arterial blood flow (0.6–4 Pa) [7]. Interestingly, the adhesion of cancer cells commonly occurs in microvasculature [8]. Therefore, the presence of DCC in microvasculature can be a key factor in altering WSS in the vicinity of DCC which can consequently influence the function of endothelial cells to express VEGF. Thus, the study of the role of DCC in altering WSS in its vicinity in a microvessel is the focus of this work.

**Fig 1:**
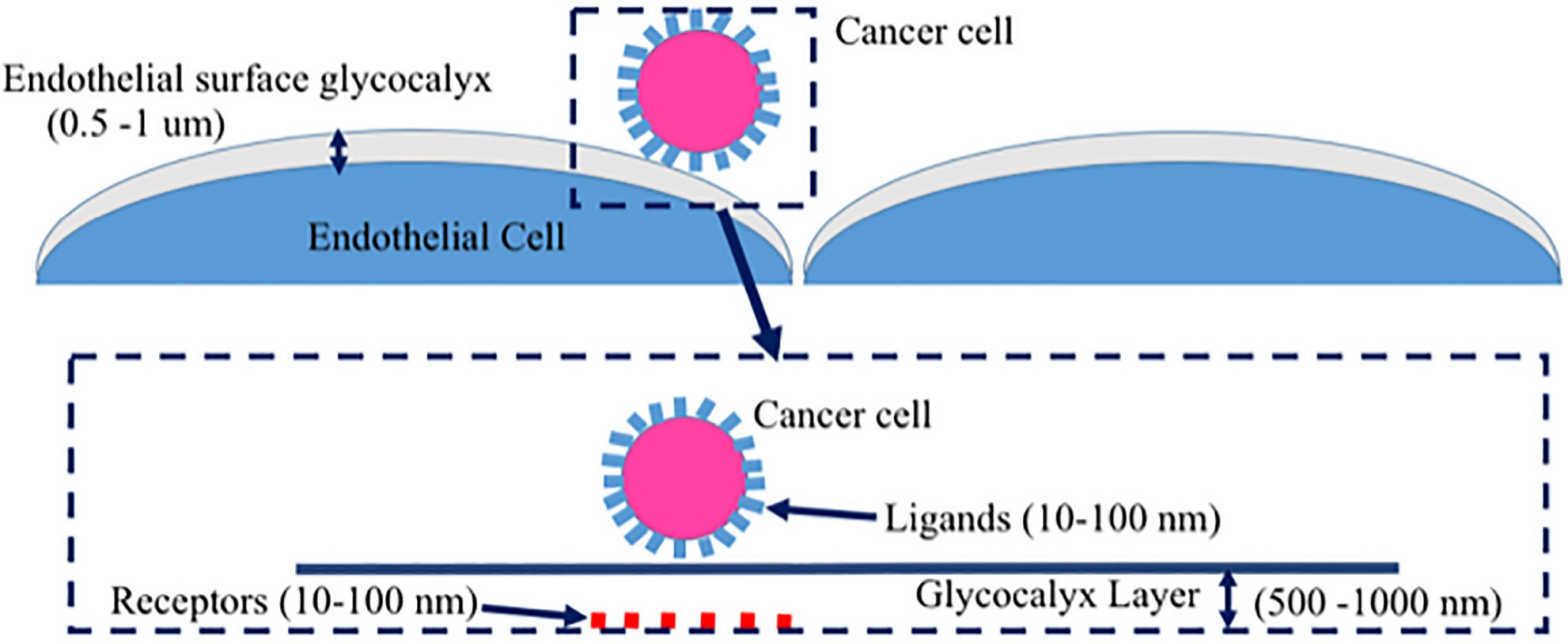
Schematic illustration of a cancer cell circulating in vicinity of endothelial surface glycocalyx. A) Cancer cell in vicinity of endothelial surface glycocalyx. B) Receptors on a substrate and ligands on a membrane. The glycocalyx layer is anchored on the endothelial cell surfaces.

Healthy glycocalyx has been recognized as a major physiological regulator of DCC adhesion to endothelium, which shields the endothelial adhesion molecules against DCC [1–5]. The thickness of glycocalyx is 0.1–1μm that is considerably larger than endothelial adhesion molecules [3–6, 9–10]. Thus endothelial adhesion receptors can be exposed by the loss of glycocalyx. Fig 1A demonstrates the schematic of a cancer cell in the vicinity of glycocalyx layer. Fig 1B depicts the schematic of receptors on a substrate along with ligands on a cell membrane. The glycocalyx layer is anchored on the endothelial cell surface (Fig 1B). It has been reported that the DCC-secreted-VEGF not only can degrade glycocalyx, but also can increase the exposure of receptors on the endothelial surface as well as the transendothelial migration of DCC through endothelium in microvasculature [3–6]. The expression of VEGF by the endothelium are significantly distinctive between arteries and veins/capillaries due to differences in WSS magnitude [7,11]. Furthermore, a previous *in vivo* study reported that most deformable cancer cells are entrapped/arrested in capillaries, post-capillary venule intersections, or post-capillary venules [8, 12]. Taken all together, it is still not clear whether endothelium plays any role in the glycocalyx degradation within the microvasculature where cancer metastasis commonly takes place. An understanding of how the presence of a DCC influences hemodynamic forces on the endothelium is needed before we can discern potential connection between the endothelium and glycocalyx degradation.

In the present study, we first investigate how a migrating DCC through microvasculature influences the hemodynamic features, particularly WSS, in its vicinity, then identify locations of the microvasculature with sufficiently high WSS on vessel wall for VEGF expression by endothelium, and finally determine parameters predisposing WSS to increase. Our study examines, for the first time, the impact of cancer cell deformability on local elevation of WSS over endothelium. We have conducted simulations with HARVEY [13–14], a massively parallel computational fluid dynamics solver based on the lattice Boltzmann method to investigate the local hemodynamics around DCC migrating within microvasculature. In this study, we leverage fluid-structure-interaction functionality implemented via the immersed boundary method to couple a finite element model for DCC with the fluid model [15]. To the best of our knowledge, no previous study investigated the hemodynamic conditions in the vicinity of migrating DCC within the microvasculature. The motion of deformable spheres in a cylinder has been extensively studied in the literature [16–29], but there are unaddressed issues that constitute our main framework in this paper as follows: a) Earlier studies have focused on exploring the effect of hemodynamics on the motion and deformation of a sphere, whereas we have diverted our attention to how a deformable sphere (cancer cell) influences the hemodynamic characteristics on neighboring regions of endothelium; b) Unlike earlier studies, we have identified the linkage between the location/ properties of the sphere and the measured WSS over the capillary wall surface; and c) We have located the sphere near the capillary wall (cancer cell margination) whereas it was located at the center of the capillary cylinder, in other studies [16–29]. We have observed, for the first time, that DCC boosts WSS in a nearby zone up to 12 Pa which is in the range to induce VEGF expression by endothelium [9–10, 30–33]. The findings of the current study shed some light on the critical role of endothelium in a cancer metastasis cascade and help to initiate accurate criteria that facilitate the cancer metastasis.

## 2. Methods

### 2.1 Lattice Boltzmann

We have conducted the simulations using HARVEY which implements the lattice Boltzmann method to conduct computational simulations of the fluid flow in complex 3D geometries [13–14, 34–36]. The lattice Boltzmann method is a deterministic, mesoscopic approach to numerically solve the Navier-Stokes equations governing fluid flows. The lattice Boltzmann method discretize the space and the velocity with a fixed Cartesian lattice, and models the fluid with a particle distribution function $f_{i\;}(\vec{x}\;,\;t)$ which denotes probability of finding a particle at time *t* and lattice point $\vec{x}$ with the discrete velocity ${\vec{C}}_{i}$. Evolution of the distribution *f* is governed by the lattice Boltzmann equation for a timestep *δt*:

$$f_{i}(\vec{x}\;+\vec{C_{i}}\;\delta t,t\;+\delta t)-f_{i}(\vec{x}\;,t\;)\;=\;-\omega \;(f_{i}(\vec{x}\;,t\;)\;-f_{i}^{eq}(\vec{x}\;,t\;)\;+\;\delta t\;F_{i}(\vec{x}\;,t\;)$$ (1)

Local equilibrium $f_{i}^{eq}(x,t)$ is a second-order expansion of local Maxwellian-Boltzmann distribution. Distribution ***F*** corresponds to an external force ***g*** which is applied to the fluid. The implementation of lattice Boltzmann method in HARVEY makes use of a standard D3Q19 velocity discretization, a $\text{BGK}\;\text{collision}\;\text{operator}\;\omega \;=\;\frac{1}{\tau }$, and a kinematic viscosity $V=C_{S}^{2}\;(\tau -\;\frac{1}{2})$ with a lattice speed of sound $C_{S}\;=\;\frac{1}{\surd 3}$.

A no-slip boundary condition is enforced on geometry surfaces by a halfway bounce-back boundary, while Zou-He boundary conditions are used at inlets and outlets with a fixed velocity profile at the inlet and a fixed pressure at the outlet [15]. Collision kernel from previous HARVEY implementations is extended to include an external force distribution **F** derived from an external force **g** [13–14, 34–36]. External force is used in the immersed boundary method to couple the dynamics of fluid and cancer cell. Hydrodynamic moments, density *ρ* and momentum *ρ***v**, in this kernel are computed as

$$\rho \;=\;\sum_{i\;=\;1}^{19}f_{i}$$ (2)

$$\rho V\;=\;\sum_{i\;=\;1}^{19}{C_{i}\;f}_{i}+\;\frac{\delta t}{2}\;g$$ (3)

Velocity **v** is used to compute a Maxwell-Boltzmann equilibrium distribution. The **v** and **g** are applied to compute components of an external force distribution.

To compute WSS vector *τˆ*, a stress tensor σ_αβ_ is computed from a non-equilibrium distribution by

$$f_{i}^{neq}\vec{(x}\;,t)\;=\;f_{i}\vec{(x}\;,t)-\;f_{i}^{eq}\vec{(x}\;,t).$$

Using the approach from Matyka et. al., [37], an outward normal vector nˆ is estimated and WSS vector components computed as *τ*_*a*_ = *σ*_*α β*_ *nˆ*_*β*_ *− (nˆ*_*β*_*σ*_*γβ*_*nˆ*_*γ*_*)nˆ*_*α*_. Additional details about HARVEY implementation, parallelization, and scaling may be found in [13–14, 34–36].

### 2.2 Cancer cell finite element model

There have been several attempts to study the effect of different parameters (e.g. membrane mechanical properties) on the cell deformation [12, 16–20, 38–44]. Systematically, cell is modeled as a two-dimensional rigid sphere [12, 38–41]. More recently, the immersed boundary method [12, 42] or the volume-of-fluid [43–44] method have been applied for three-dimensional simulations of circulating cells. These methods have generally modeled the cells as fluid-filled capsules surrounded by a single zero-thickness [16–17] or finite thickness membranes [18]. Luo et al applied a front-tracking method to study the deformation of an elastic compound capsule in a shear flow [19–20]. These results were extended from initially spherical to ellipsoidal compound capsules where the dynamics of cell deformation in a shear flow was investigated in depth in [20]. We followed the approach described by Luo *et al*. [19–20], to represent a cancer cell as a compound capsule with zero-thickness membrane to represent an outer cell membrane. Major cellular elements are incorporated in the model including an outer cell membrane and a cytoplasm. The cell membrane is numerically described by 20480 flat triangular face elements [21]. The triangulated grid for the membrane is created by taking successive subdivisions of an icosahedron, which produces a favorable regularity and isotropy, and projecting onto the cell initial spherical shape [15, 21]. Shear and dilational elastic responses to a strain of cell membrane are governed by a Skalak constitutive law,

$$W\;=\;\frac{G}{4}(I_{1}^{2}+2I_{1}-2I_{2}+CI_{2}^{2})$$ (4)

Where *I*_*1*_, *I*_*2*_ are strain invariants, E is shear elastic modulus, and *C* is ratio of dilational to shear elastic moduli (*C* = 1 to model the cell membrane as an area compressible biological membrane) [21]. A simple finite element model is used to compute membrane forces G from a strain energy function [22]. A penalty force *f*_*p*_ is applied to enforce a constant cell volume; the resulting volume variation is less than 1% for the cell membrane during all subsequent simulations. Moreover, we took into account the membrane bending resistance with a bending modulus of K_b_ = 2 × 10^−18^ J [25–26, 40–45]. The cytoplasm and the plasma are assumed as Newtonian fluid [37]. We have previously shown that [15] the deformed shape of cells does not change significantly with time. Thus, our method focuses on a quasi-steady motion of the cell where the cytoplasm viscosity has negligible effect on the dynamic of cell. We therefore apply a same viscosity of *μ* = 1.2 ×10^−3^ Pa.s for the plasma and the cytoplasm [37].

### 2.3. Immersed boundary

Fluid-solid interaction between the cancer cell and the fluid are performed by coupling the finite element model for the cell (defined on a Lagrangian grid) with the lattice Boltzmann method (defined on a Eulerian grid) [24]. A discrete delta function δ is used to transfer physical values between the two grids. The immersed boundary method enforces a no-slip condition on the capsule surface and allows the cell to exert a force on the surrounding fluid. In our implementation, there are three components of the immersed boundary method: spreading, interpolation, and updating.

The force **G** defined on the cell vertex **X** is spread onto the fluid grid by the equation

$$g(x,t)\;=\;\sum_{x}G(X,t)\delta (x-X(t))$$ (5)

The fluid velocity **v** is updated with the lattice Boltzmann algorithm. In the interpolation step, the updated velocity **V** of the capsule vertex **X** is computed at time t + δt with the sum

$$\text{V}\left(X,t+\delta t\right)=\sum_{x}v\left(x,t\right)\delta \left(x-X\left(t\right)\right)$$ (6)

Finally, the position of capsule vertex is updated using a no-slip condition:

$$X_{\left(t+\delta t\right)}=X_{\left(t\right)}+\;V_{\left(t+\;\delta t\right)}$$ (7)

In this implementation, a discrete delta function $\delta (x-X(t))\;=\;\prod_{i\;=\;1}^{3}\delta_{i}\;(x_{i}\;-\;X_{i})$ is defined by a one-dimensional delta function

$$\delta_{i}\left(r\right)=\{\begin{array}{ll} \frac{1}{4\delta x}\left(1+\text{cos}\left(\frac{\pi r}{2\delta x}\right)\right) & r\leq 2\delta x\\ 0 & r>2\delta x \end{array}$$ (8)

using a spatial step δx of the fluid grid and *r* = (*x*_*i*_ − *X*_*i*_)*δx*^−1^. A general discussion of the coupling of lattice Boltzmann method and finite element methods with the immersed boundary method may be found in [16]. In this study, the mesh size of lattice Boltzmann method was set to be 150 nm, and that of the finite element model was approximately 150 nm (an unstructured mesh with 20480 elements for cancer cell).

### 2.4. Model parameters

Test ranges and reference values of the geometric and mechanical parameters of the DCC and the capillary applied in our model are summarized in Table 1. Table 2 includes all symbols. The time step is set to 6.7 ns corresponding to a grid resolution size of 150 nm. Data are analyzed at t = 1 ms, prior to the formation of adhesive bonds between the cancer cell and endothelium.

**Table 1:** Model parameters in the simulations.

Definition	Parameters	Value	Reference
Cancer cell diameter	Dc	8 μm	[12, 15, 38]
Capillary diameter	Dv	8.82, 9, 10, 10.4, 12, 14, 16 μm	[12, 46]
Cell surface shear elastic modulus	E	30, 3, 0.3 *μ*N/m	[12, 15, 23, 37, 46]
Bending modulus	K_b_	2 × 10^−18^ J	[18, 32–33]
Plasma density	*ρ*	1 × 10^3^ *kg*/*m*^3^	[12, 15, 38]
Cytoplasm viscosity	*μ*	1.2 × 10^−18^ Pa.s	[12, 15, 38]

**Table 2:** List of symbols.

DCC	deformable cancer cells
WSS	wall shear stress
VEGF	vascular endothelial growth factor
Rv	vessel radius
A_aff_	characteristic affected area
δx	spatial step of the fluid grid
Ca	capillary number
γ	shear rate
Rc	outer membrane radius

## 3. Results

### 3.1. Validation

**Fig 2:**
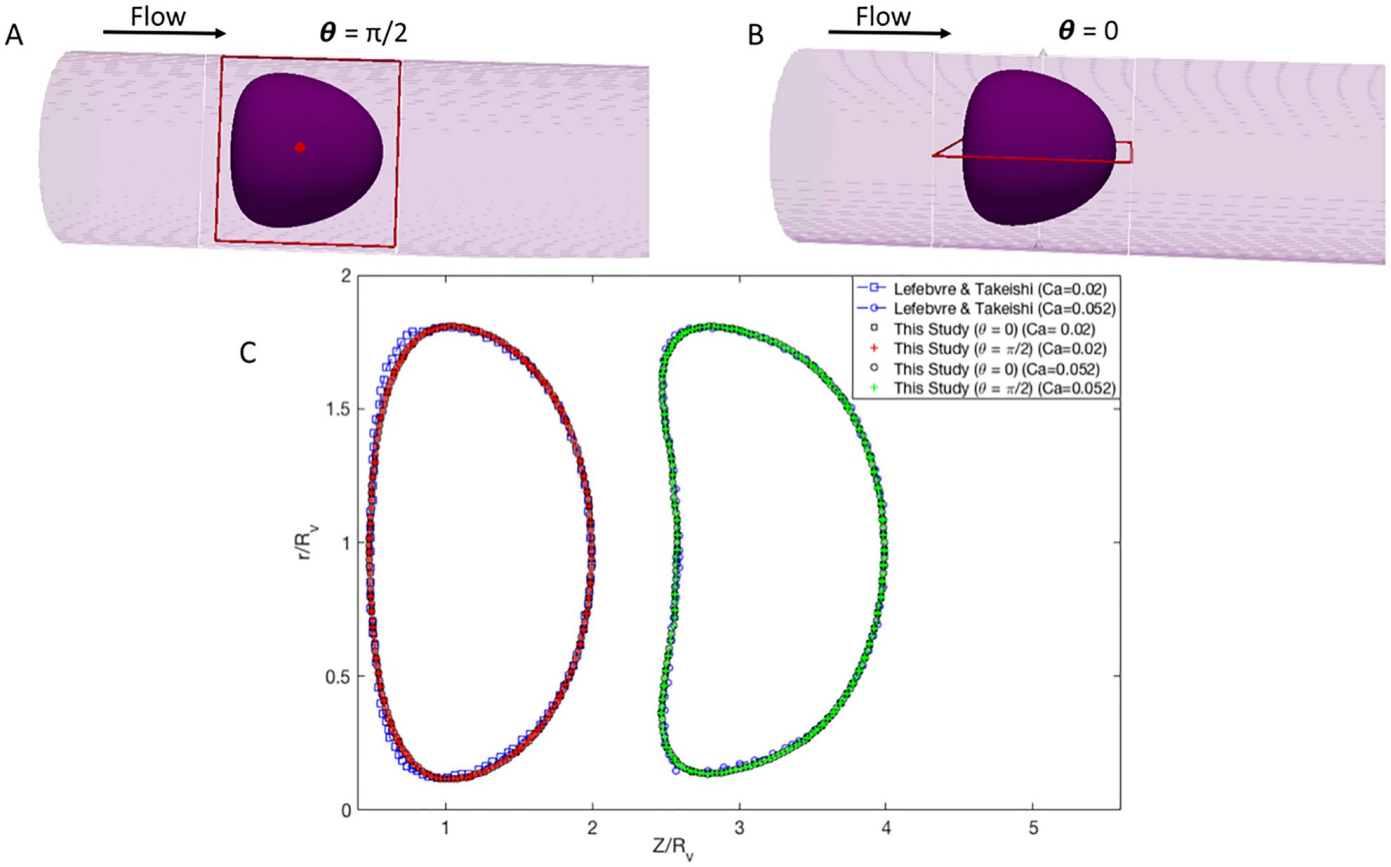
Comparison between current results and Lefebvre at el., for the shape of deformable, cancer cell migrating freely in a capillary when Dc/Dv = 0.77. Note that cell is located at the center of the capillary. Rv stands for the vessel radius. Z is the flow direction.

Our fluid-solid interaction framework is validated with the results of Takeishi N et al. [12] and Lefebvre et al. [25], by comparing the deformation of an initially spherical cancer cell located at the center of a vessel with the diameter of 10.4 μm (shown in Fig 2A and 2B). The DCC is freely moving with the fluid in the capillary where the ratio of cell diameter (Dc) to vessel diameter (Dv) is 0.77. The deformation of DCC is examined under capillary numbers as Ca = 0.02 and 0.052, demonstrated in Fig 2C. Ca is defined as μγRc/E, for shear rate γ and outer membrane radius Rc. γ is defined as γ = U/Dv, where U is the fluid velocity at the inlet. Here, we have applied similar mechanical properties for the DCC as in Ref. [12]: the surface shear elastic modulus is set to 30 *μ*N/m and the viscosity of plasma and cell cytoplasm is set to 1.2 × 10^−3^ Pa.s. It is evident from Fig 2C that our results on the cell deformation are in good agreement with the results reported in Ref. [12] and Ref. [25]. Furthermore, to determine an optimized grid spacing, we have compared the shape of DCC to the simulations of Refs. [12 and 25]. Consistency in cell deformation is observed in the grid spacing of 125, 140, 150, 160, and 180 nm. We set the grid spacing to 150 nm in this study. Fig 2C shows the deformed shape of cell under Ca = 0.02 and 0.052, with the grid resolution size of 150 nm.

### 3.2. Migration of deformable, circulating cancer cell within the capillary

In this part, we investigate the migration of DCC, the deformation of DCC, and the impact of DCC on its local hemodynamics. Three distinctive values of surface shear elastic modulus, E, (i.e. 30 *μ*N/m, 3 *μ*N/m, and 0.3 *μ*N/m) are examined for the DCC [8, 12, 25, 42, 47]. The migration of DCC within vessels with different diameters as 8.82 *μ*m (Dc/Dv = 0.907), 9 μm (Dc/Dv = 0.889), 10 μm (Dc/Dv = 0.8), 12 μm (Dc/Dv = 0.667), 14 μm (Dc/Dv = 0.571), 16 μm (Dc/Dv = 0.5) is studied [8, 12]. The range of capillary diameter is chosen based on findings of Ref.[4] on the localization of deformable cancer cell adhesion in the microvasculature [4,5,8]. Note that the DCC is initially located by microvilli whose average length is 0.5μm [12, 27].

**Fig 3:**
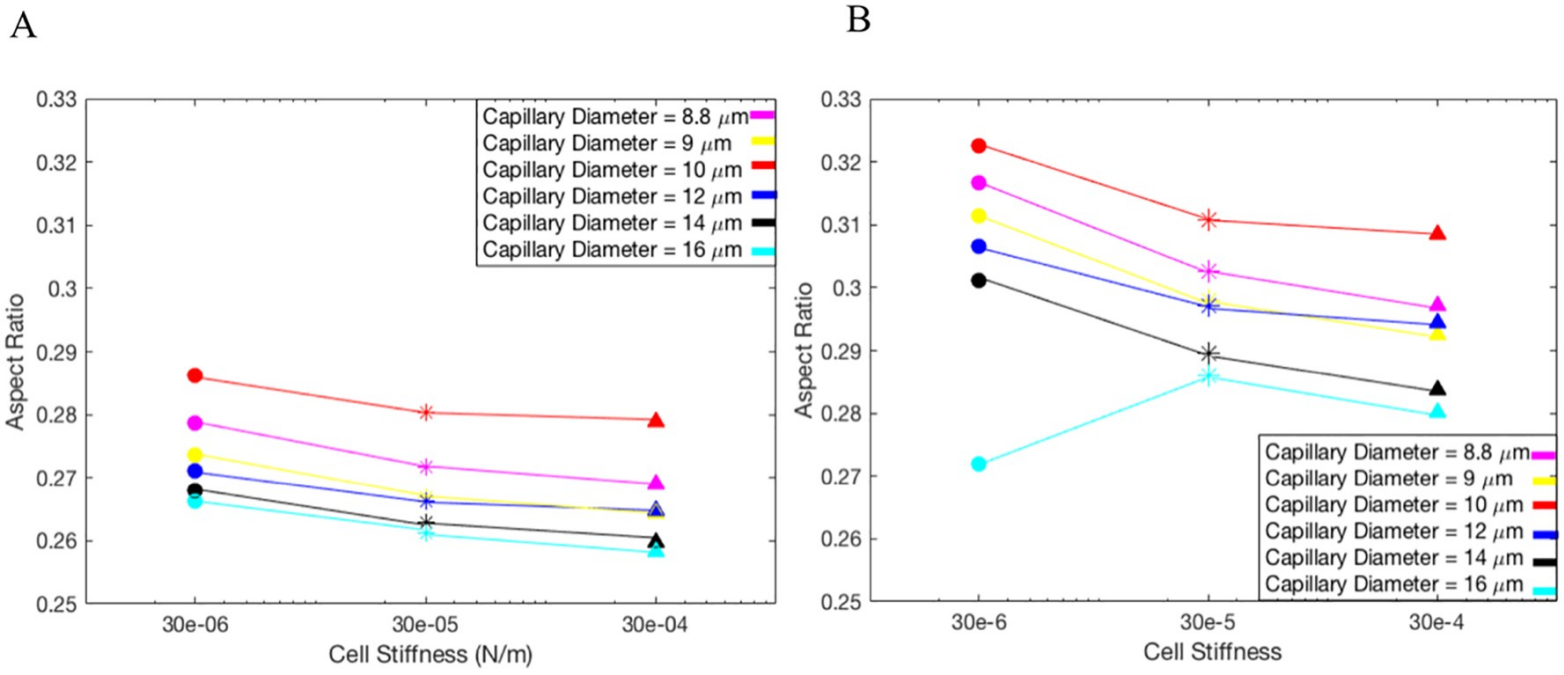
Aspect ratio of a deformable cancer cell migrating within microvasculature. The diameter of capillaries varies in the range of 8.82–16 μm. A) γ = 125 s^-1^. B) γ = 312 s^-1^.

**Fig 4:**
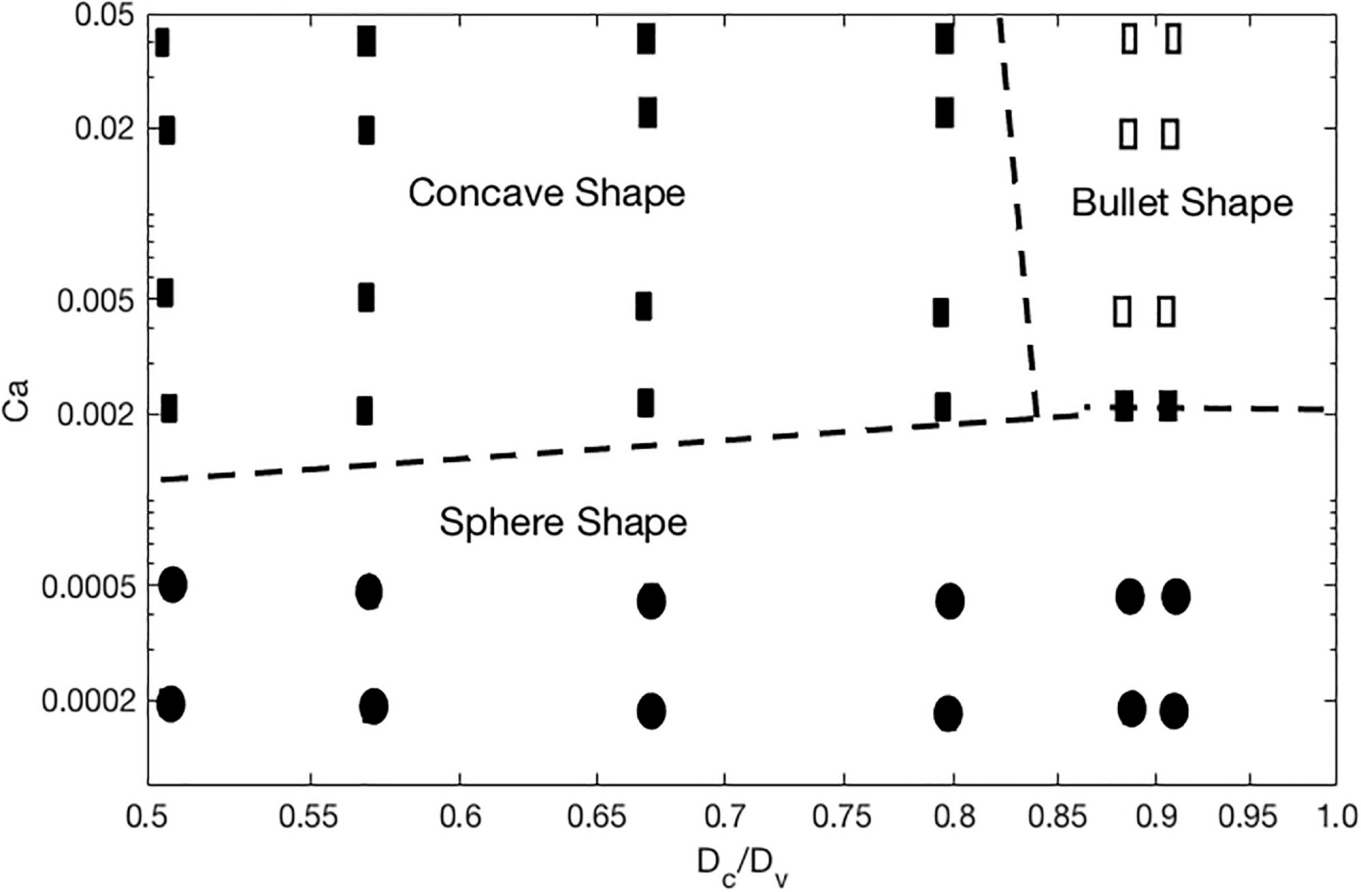
State diagram of a deformable cancer cell motion in capillaries as the function of capillary number Ca and normalized capillary diameter (Dc/Dv). Filled squares represent rolling motion of the cell and open squares correspond to bullet motion.

**Fig 5:**
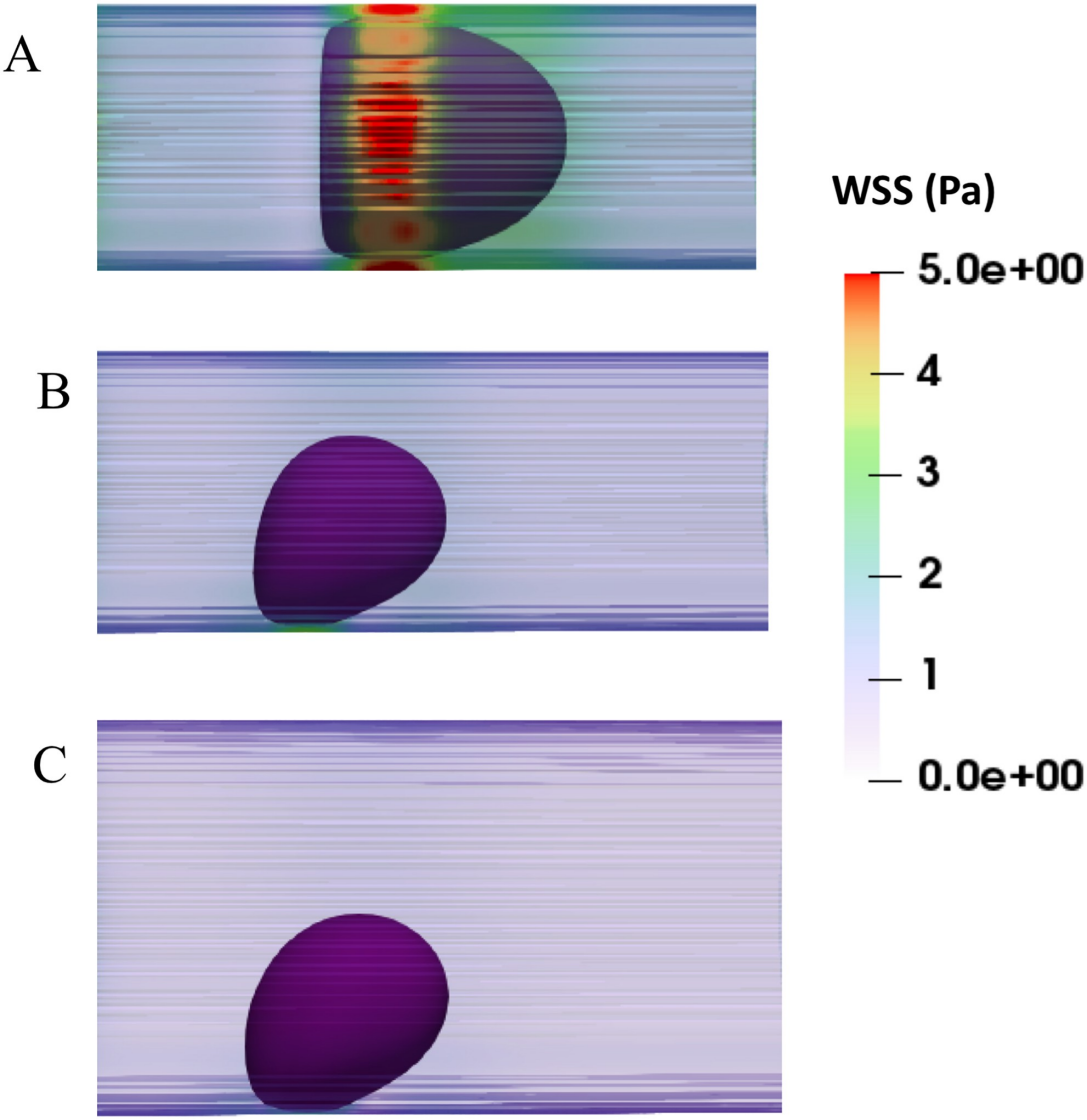
The distribution of WSS over endothelium. A) Dc/Dv = 0.907 and γ = 312 s^-1^. B) Dc/Dv = 0.667 and γ = 312 s^-1^. C) Dc/Dv = 0.5 and γ = 312 s^-1^. Note that shear elastic modulus of the cancer cell is 30 N/m in all panels of Fig 5.

**Fig 6:**
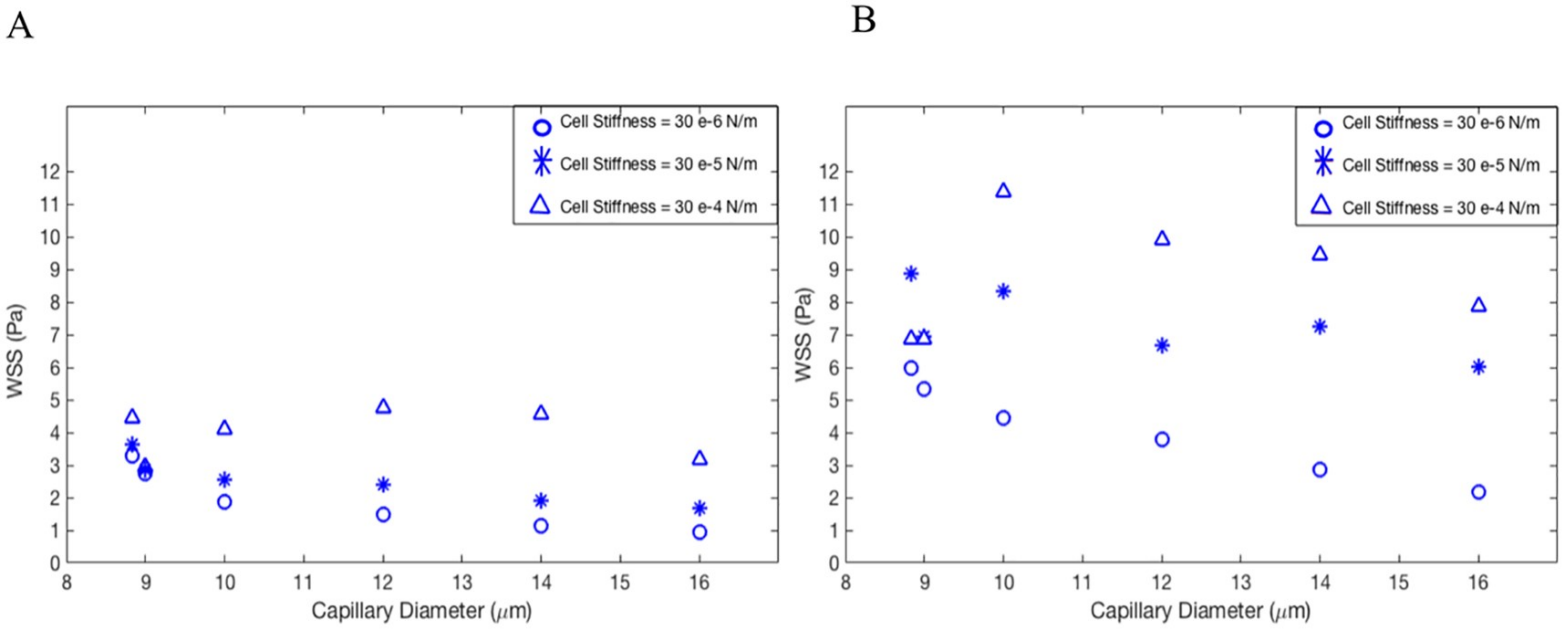
Maximum magnitude of WSS over endothelium in the vicinity of circulating cancer cell. Cells with different stiffnesses are migrating within capillaries with diameters varying in the range of 8.82–16 μm. A) γ = 125 s^-1^. B) γ = 312 s^-1^.

**Fig 7:**
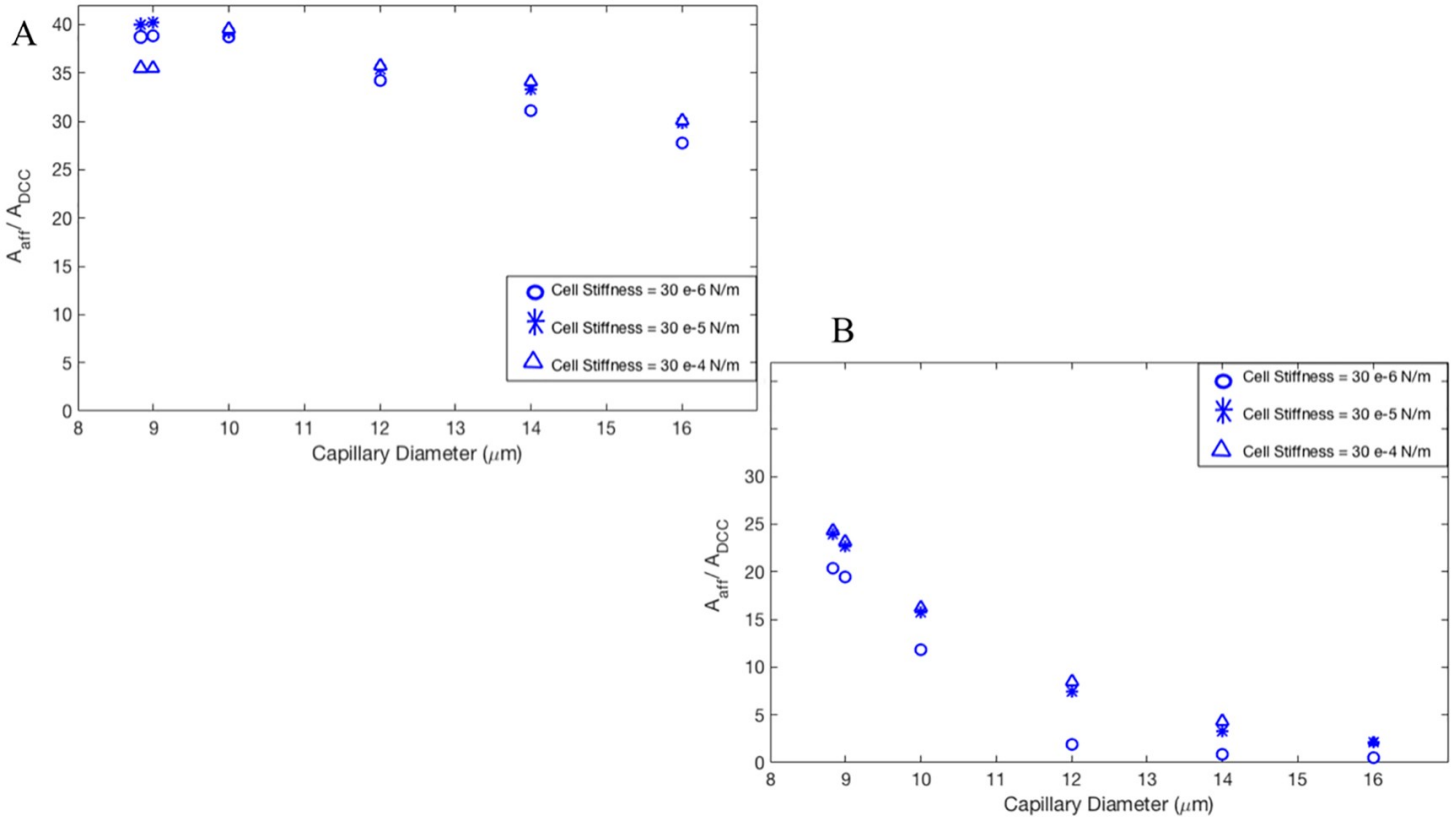
Local changes to hemodynamics nearby a deformable, circulating cancer cell. The area of endothelium, A_aff_, (characteristic affected area) in the vicinity of cancer cell (with WSS > 0.6 Pa) is demonstrated. A_DCC_ is lateral surface of the cancer cell. Note that cancer cells with different stiffnesses are migrating within capillaries with diameters varying in the range of 8.82–16 μm. A) γ = 125 s^-1^. B) γ = 312 s^-1^.

Fig 3 shows the deformation of DCC which is measured using Taylor deformation parameter of aspect ratio = L-B/L+B, where L and B are major and minor axes [15, 19–20]. Fig 3A presents the aspect ratio versus Dv for three different cell shear elastic moduli and γ = 125 s^-1^[12, 47]; whereas Fig 3B corresponds to γ = 312 s^-1^[12, 47]. It is evident from Fig 3 that the deformation of cancer cells varies significantly with the diameter of capillary, mechanical properties of DCC, and hemodynamic conditions within the capillary. As seen in Fig 3, the aspect ratio drops with increasing the capillary diameter, but largest magnitude of aspect ratio is observed at Dv of 10 μm shown in both Fig 3A and 3B. This biphasic behavior can be explained by looking at the motion of DCC in different capillary. A state diagram of the cancer cell motion is demonstrated for various capillary size and the capillary number Ca in Fig 4. For Dc/Dv > 0.8, the DCC exhibits a bullet-like motion for Ca > 0.002 and a sphere-like motion for Ca ≤ 0.002. The deformation of DCCs to bullet-like shape has also been previously observed by Takeishi N et al [12] in the subset of vessels whose diameter is comparable to cell diameter. For Dc/Dv ≤ 0.8, the cell always exhibits a concave shape for Ca ≥ 0.002 and a sphere shape for Ca ≤ 0.002. The deformation of DCCs to concove shape has also been previously observed by Zhang et al [47] in the subset of vessels whose diameter is larger than the cell diameter. At the Dv of 8.82 and 9 μm, the cell exhibits bullet-like shape or sphere shape (depending on the cell stiffness), which are both symmetric shapes. The symmetry is a direct result of the flow field in the gap between the cell and the capillary wall. As the diameter of capillary increases, the gap area becomes asymmetric exposing the cell to a higher shear rate in the direction of larger gap. Therefore, around the Dv of 10 μm, the cell deforms asymmetrically resulting a concave shape. However, further increase in the capillary diameter increases the gap with lowering the shear rate, which will reduce the deformation of the concave shape. Furthermore, the biphasic behavior observed in Fig 3B for Dv of 16 μm can be explained by the fact that the cell stiffness characterizes the time scale of deformation experienced by the flow shear rate. The cell deformation time scale competes with the time scale of the flow in the gap. If the former is smaller, the cell will tend to relatively maintain its shape as spherical, which leads to a smaller aspect ratio. Fig 5 demonstrates the distribution of WSS over the endothelium in capillaries with three different diameters. Moreover, Fig 5 reveals the connection between the cell distance to the vessel wall and WSS exerted by the DCC on endothelium. It is evident from Fig 5 that the magnitude of WSS is significantly discrepant at two sides of the vessel wall with higher magnitudes observed at the side of vessel which is closer to the DCC. Fig 6 displays the maximum of WSS in the vicinity of the DCC. Fig 6 elaborates several critical findings about how the stiffness of DCC, hemodynamic conditions inside the capillary, and the capillary size can control the magnitude of WSS over endothelium. The findings can be listed as follows: A) endothelium tolerates a higher magnitude of WSS nearby a stiffer DCC; B) DCC’s stiffness has a higher impact on WSS for capillaries with the diameter > 9μm; C) for capillaries with the diameter < = 16μm, endothelium experiences higher WSS (> 0.6 Pa) independent of the DCC stiffness and Ca. The area of endothelium affected by the DCC is quantified and shown in Fig 7. This area, A_aff_, (characteristic affected area) is specified by WSS > 0.6 Pa [4] and is divided by the lateral surface of the DCC (with diameter of 8μm). Moreover, Fig 7 demonstrates the influence of DCC stiffness and the anatomical structure of capillary on A_aff_ for: A) γ = 125 s^-1^, and B) γ = 312 s^-1^.

## 4. Discussion

Circulating, deformable cancer cells facilitate their own adhesion to the endothelium by secretion of VEGF that locally degrade the glycocalyx layer, resulting in the exposure of endothelial adhesion molecules [3,5–7]. In addition, high local shear stress has been shown to be a powerful stimulus for VEGF expression by endothelial cells [9, 48– 49]. However, mechanisms underlying the expression of VEGF by endothelial cells of microvasculature, where the adhesion of cancer cells usually occurs, have not been studied yet. Our study, for the first time, investigates the migration of a DCC in a capillary to explore how the presence of DCC alters hemodynamic conditions in its neighborhood especially the resultant local shear stress on the endothelium. We have applied a computational method that couples the finite element model for deformable cancer cells with the fluid model. We have examined the role of DCC mechanical properties and DCC location on the changes in local hemodynamic conditions. Our results reveal that the anatomical structure, cell stiffness, and hemodynamic characteristics of microenvironment dictate the deformation of DCC (Figs 3, 4, 5 and 6) which will change in turn the hemodynamics in its surrounding area (Figs 5, 6 and 7). Our computational study reveals that the DCC alters the local hemodynamics significantly in capillaries with Dc/Dv > 0.5 (Figs 5, 6 and 7). We also show that the stiffer DCC (shear elastic modulus > = 3 *μ*N/m) imposes a high WSS (> 0.6 Pa) on the endothelium in larger areas.

Endothelial cells experience a high WSS (> 0.6 Pa) in the vicinity of DCC up to an area as large as 40 times of the DCC lateral surface area (Fig 7A and 7B), whereas they experience WSS > 1.5 Pa in an area about 25 times of the DCC lateral surface. In the capillary with Dc/Dv = 0.5, the influenced area is 0.6 times the DCC lateral surface area corresponding to a distance of 2.5 DCC diameter, when DCC has the surface shear elastic modulus of 30 *μ*N/m and γ = 125 s^-1^ (Fig 7A). This result is consistent with the findings of Ref. [50] which developed a mathematical model to quantify the influence of neutrophil migration in the parallel-plate flow chamber. Reference [50] reported that neutrophils may influence the flow stream in its vicinity up to a distance of 2.5 cell diameter away, when γ = 125 s^-1^. On other hand, the range of WSS > 0.6 Pa has been reported to regulate the secretion of VEGF by endothelial cells [4–9, 48, 51, 30–33]. White et al. [30] evaluated effect of elevated shear stress on endothelial behavior and reported that WSS > 1.5 Pa induced a specific change in behavior, modified gene expression, reduced reactive oxygen species levels, altered MAP kinase signaling, reduced cAMP levels, and enhanced VEGF expression. These influences were all upregulated significantly by WSS > 7.5 Pa compared to WSS > 1.5 Pa [30]. Our results demonstrate that WSS > 7.5 Pa occurs in the vicinity of stiffer cancer cells migrating in all capillary sizes under the shear rate of 312 s^-1^ while softer tumor cells expose WSS > 7.5 Pa in their vicinity only in capillaries with Dc/Dv > 0.5 and under the shear rate of 312 s^-1^.

A previous study investigated the time- and dose-dependent upregulation of endothelial VEGF expression by the fluid shear stress in microvascular and large-vessel derived endothelial cells [48]. Western blot analyses performed by Ref. [48] revealed a substantial increase in VEGF protein expression as early as 6 hours for endothelial cells exposed to WSS of 1.5 Pa. Ref.[48] reported a significant increase, in a significantly shorter time course, in the expression of VEGF by endothelial cells exposed to WSS of 4.5 Pa. Moreover, several previous studies have focused on the shear stress-dependent endothelial protein expressions by examining the association between the onset of elevated shear stress and the activation of signal transduction pathways [31–33]. Prasad et al. [31] examined the biological response of endothelial cells to the elevated shear stress for periods ranging from 15 seconds to 24 hours. Ref. [31] reported that the induction of 3 Pa shear stress was associated with an early, transient but significant increase (142%) in endothelial cells response at 15 seconds of the shear stress exposure followed by a major peak (189%) observed in 5 minutes. Refs. [32 and 33] reported that the shear stress of 1 Pa induces a rapid (within milliseconds to few seconds) induction of VEGF by endothelial cells. In the present study, the DCC migrates with velocities in the range of 1 × 10^−4^ m/s (γ = 125 s^-1^) to 2 × 10^−4^ m/s (γ = 312 s^-1^). We demonstrate in Fig 7A and 7B that the DCC influences endothelium in an area up to 40 times its lateral surface area (approximately 310 *μm*). Therefore, the exposure time of endothelial cells to a high shear stress may be sufficiently long for VEGF expression by endothelium in the vicinity of the DCC, particularly in capillaries with Dc/Dv > 0.5 [30–33, 48].

We have focused on the stage prior to the formation of any adhesive bond between the DCC and endothelium. This stage is a particularly important stage because it will shape subsequent interactions between the DCC and endothelium. To initiate an interaction with endothelium, the DCC must first overcome the glycocalyx layer which is considerably thicker (0.1–1μm) than endothelial adhesion molecules and receptors. Thus, endothelial adhesion receptors are exposed by the loss of glycocalyx. Findings of our study show that the DCC can increase the WSS to sufficiently high range in its vicinity which can ultimately regulate the function of endothelial cells. WSS-induced-VEGF expression by endothelial cells will result in the local degradation of glycocalyx layer. The present study reveals the vital role of endothelial cells in the tumor cell metastasis cascade in microvasculature, particularly in capillaries with Dc/Dv > 0.5. Moreover, our study identifies the locations of microvasculature with sufficiently high WSS on vessel wall for VEGF expression by endothelium. Furthermore, our study establishes accurate criteria predisposing WSS to increase. The ability to predict the location in microvasculature where the degradation of glycocalyx occurs may help to anticipate the vascular regions where the extravasation of cancer cells through endothelium is most likely to occur. There are limitations to our study. Although previous experiments have shown that elevated WSS can trigger elevated expression level of VEGF, more experimental data are needed to understand in detail the interactions between the WSS and the expression of VEGF. Moreover, the current study may be farther extended to consider the impact of cancer cell size and geometry on the local hemodynamic in its vicinity. In addition, the geometry of microvasculature may influence the local hemodynamics which will be investigated in detail in our next study.

## 5. Conclusion

In the present study, we have investigated the mechanisms underlying wall shear stress-associated-VEGF secretion by endothelium which may contribute to the degradation of the glycocalyx layer in the stage prior to the adhesion of tumor cells to microvessel walls. Our findings suggest that wall shear stress imposed on endothelial cells in the vicinity of a migrating cancer cell may be introduced as a key component, alongside the cancer cell stiffness and vascular anatomical structure in regulating the adhesion of cancer cell to microvasculature; a critical process in metastatic scenario. We propose a cascade initiated by margination of a cancer cell and then changes in local hemodynamics in the cell vicinity, which alter the function of endothelial cells in these regions. VEGF then is available to increase the endothelial permeability by the degrading of glycocalyx layer which will lead to the exposure of endothelial surface receptors, ultimately facilitating the adhesion of cancer cells and the extravasation of tumor cells. Our results suggest that the reinforcing of glycocalyx integrity along with the preservation of endothelial structural integrity could be an effective treatment strategy to suppress the metastasis of cancer cells.
